# Supplementary material for: Circadian clock dysfunction in human omental fat links obesity to metabolic inflammation
Source: Nat Commun. 2021 Apr 22;12:2388. doi: 10.1038/s41467-021-22571-9 (PMC8062496; doi:10.1038/s41467-021-22571-9)
Supplement: Supplementary file 1 — Supplementary Information [file 41467_2021_22571_MOESM1_ESM.pdf]

Gene expression in omental adipose tissue from obese and non-obese patients

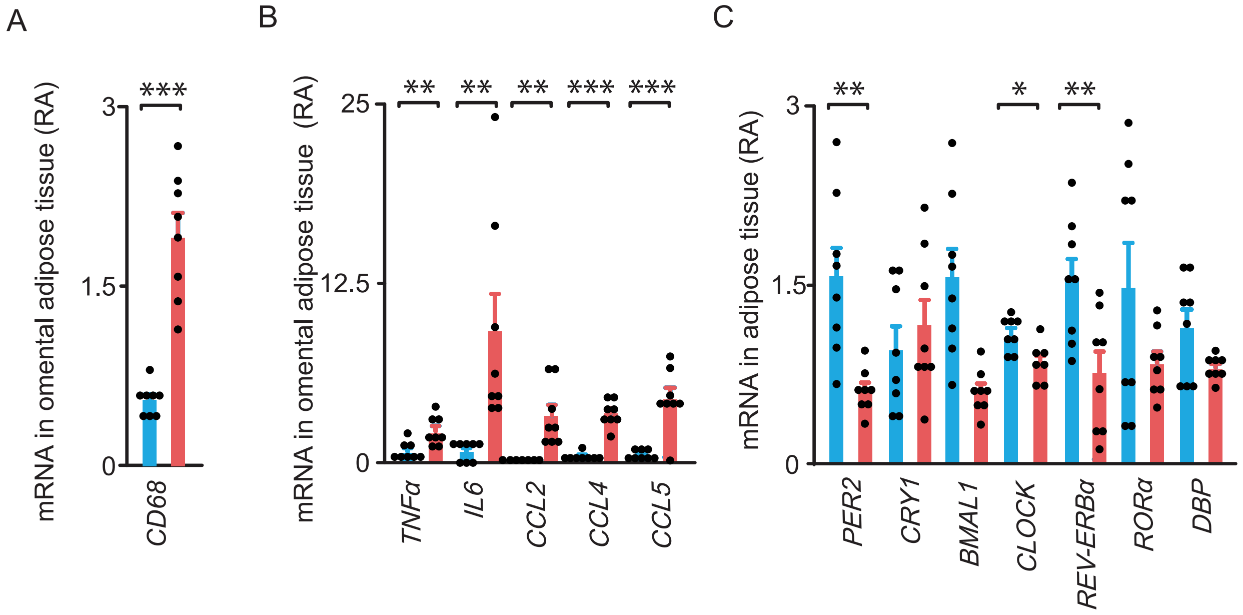

Gene expression in omental vs. subcutaneous adipose tissue from obese patients

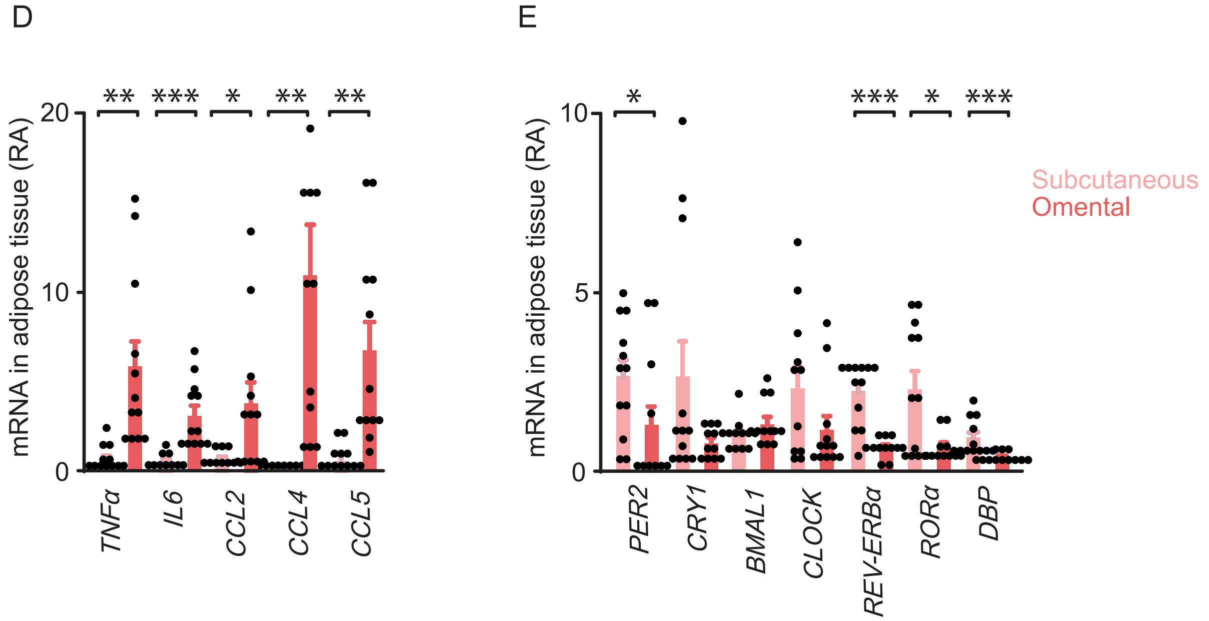

Supplementary Figure 1. Altered inflammatory and clock gene expression in human obesity
(related to Fig.1)

(**A**-**C**) QRT-PCR analysis for *CD68*, a marker of macrophages, (**A**), inflammation (**B**), and core clock components (**C**) in whole (undigested) omental adipose tissue obtained from non-obese (blue) or obese (red) patients (n = 8 patients/ group; cohort 1); *p<0.05, **p<0.01, ***p<0.01, unpaired two-tailed t-test.

(**D**, **E**) QRT-PCR measurement of pro-inflammatory (**D**) and core clock gene expression (**E**) in whole omental (red) or subcutaneous adipose tissue (pink) from obese patients (n = 12 patients; cohort 2, with the exclusion of one outlier from *CRY1* and *RORα* analyses, as determined by Grubbs' test); *p<0.05, **p<0.01, ***p<0.01, paired two-tailed t-test. Values are displayed as mRNA RA. Data are represented as mean ± SEM.

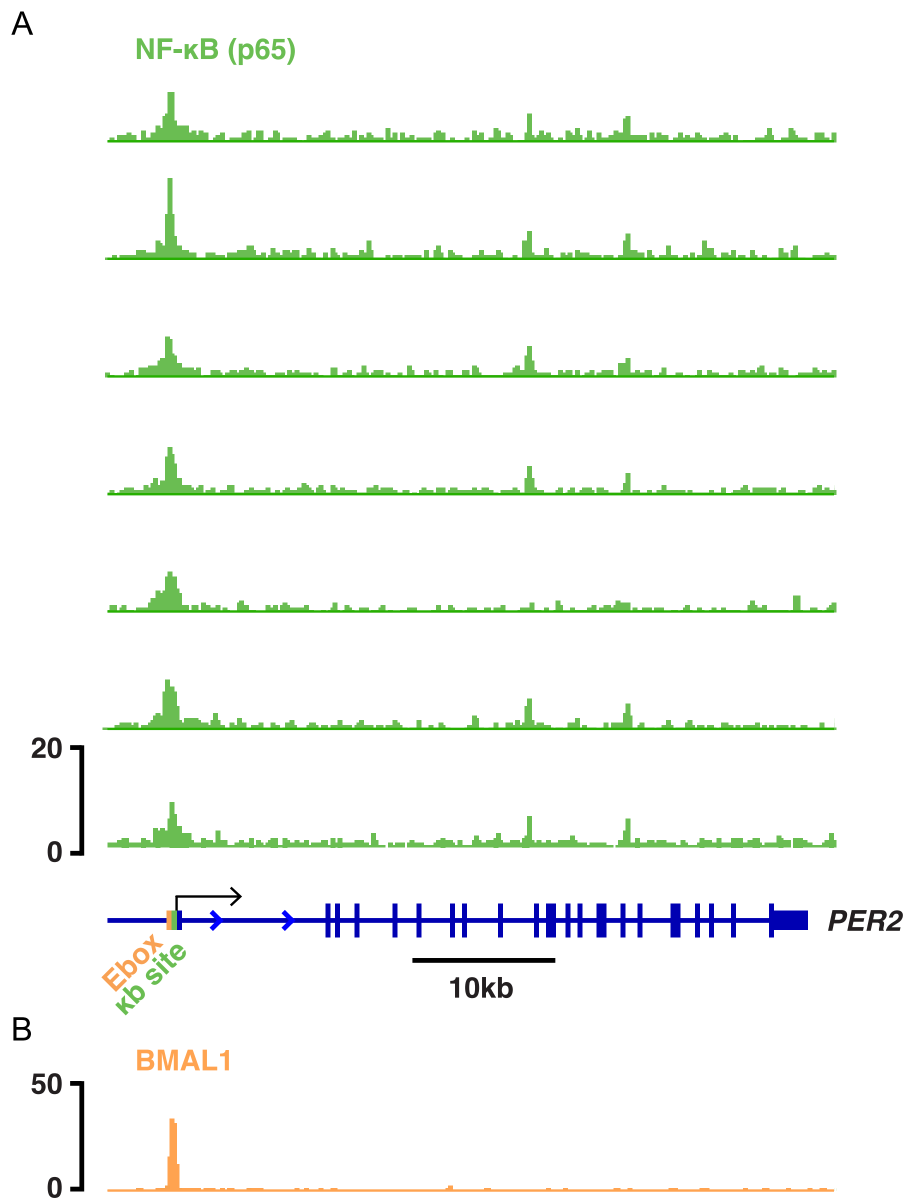

<u>Supplementary Figure 2. Close localization of NF-κB (p65) and BMAL1 peaks on *PER2* in human cells</u> (related to Fig. 2)

Overlaid Integrative genome viewer (IGV[1,2]) images of p65 (green) and BMAL1 (orange) ChIP-seq tracks from human cells, including (**A**) 7 lymphoblastoid cell lines (green) and (**B**) 1 osteosarcoma cell line (orange) are shown. Track heights are normalized to the number of aligned reads. ChIP-seq tracks are obtained from published data[3,4].

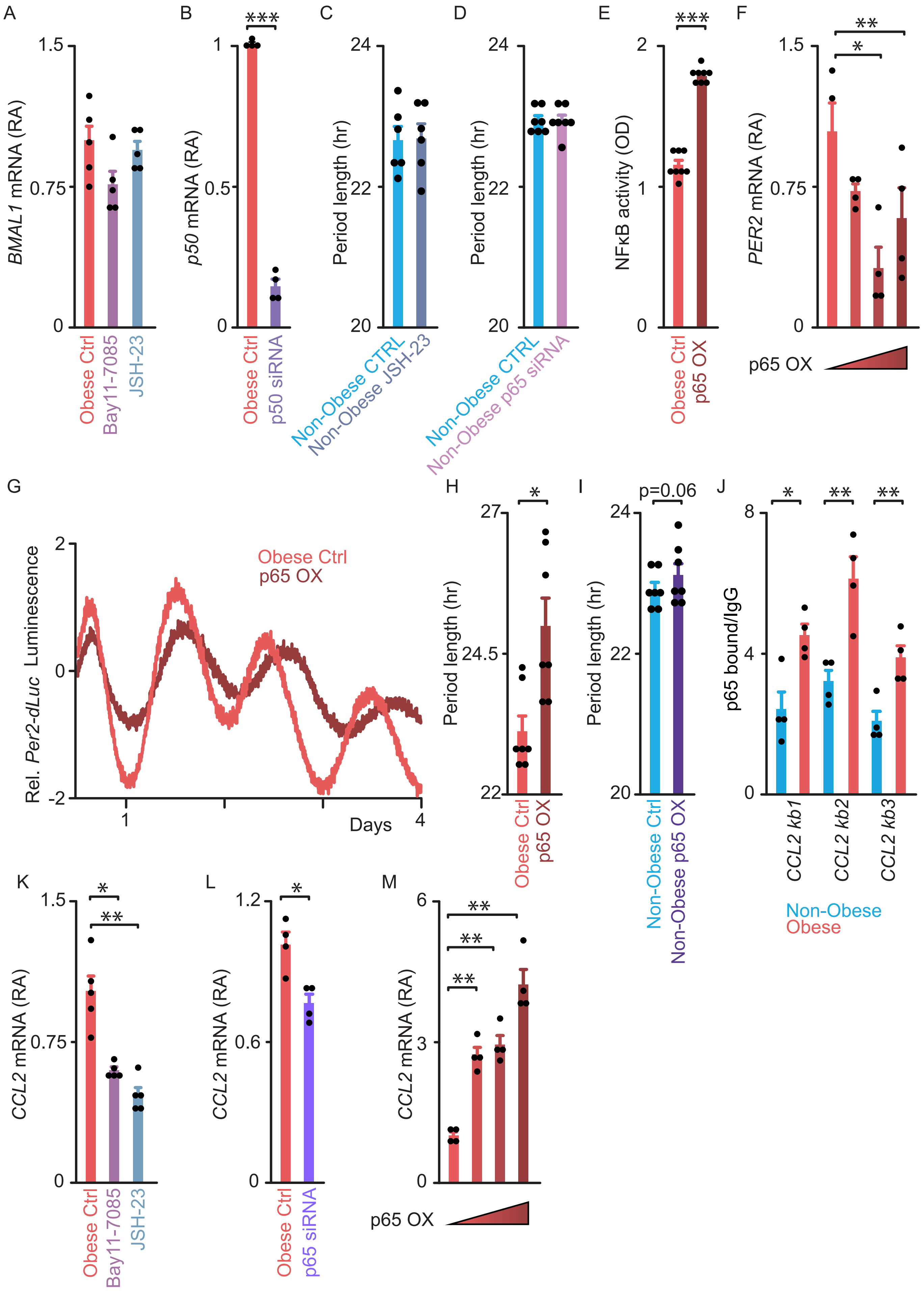

A
B
C
D
E
F
1.5
0.75
0
BMAL1 mRNA (RA)
Obese Ctrl
Bay11-7085
JSH-23
***
1
0.5
0
p50 mRNA (RA)
Obese Ctrl
p50 siRNA
24
22
20
Period length (hr)
Non-Obese CTRL
Non-Obese JSH-23
24
22
20
Period length (hr)
Non-Obese CTRL
Non-Obese p65 siRNA
***
2
1
0
NFκB activity (OD)
Obese Ctrl
p65 OX
**
*
1.5
0.75
0
PER2 mRNA (RA)
p65 OX
G
2
0
-2
Rel. Per2-dLuc Luminescence
Obese Ctrl
p65 OX
1
Days
4
H
*
27
24.5
22
Period length (hr)
Obese Ctrl
p65 OX
I
p=0.06
24
22
20
Period length (hr)
Non-Obese Ctrl
Non-Obese p65 OX
J
*
**
**
8
4
0
p65 bound/IgG
CCL2 kb1
CCL2 kb2
CCL2 kb3
Non-Obese
Obese
K
*
**
1.5
0.75
0
CCL2 mRNA (RA)
Obese Ctrl
Bay11-7085
JSH-23
L
*
1.2
0.6
0
CCL2 mRNA (RA)
Obese Ctrl
p65 siRNA
M
**
**
**
6
3
0
CCL2 mRNA (RA)
p65 OX

Supplementary Figure 3. Impact of NF-κB signaling on clock function in non-obese OAPs and on *BMAL1* and *CCL2* expression in obese OAPs (related to Fig. 3 and Fig. 4)

(**A**) QRT-PCR measurement of *BMAL1* expression in non-synchronized obese OAPs after targeted inhibition of NF-κB activity. Cells were either untreated or treated for 2hr with 10 μM NF-κB inhibitors Bay11-7085 (purple) or JSH-23 (grey) *vs*. vehicle (Ctrl, DMSO, red). Values are displayed as mRNA RA compared to values of untreated OAPs (n = 5 patients).

(**B**) QRT-PCR measurement of *p50* expression in non-synchronized obese OAPs transfected with either siRNA against human *p50* (dark purple) or non-targeting siRNA (red). Values are displayed as mRNA RA compared to values of Ctrl cells with n = 4 independent cultures per condition, obtained from 2 patients.

(**C**-**D**) Normalized bioluminescence of *Per2-dLuc* reporter oscillations in synchronized non-obese OAPs. (**C**) Cells were either untreated (DMSO, blue) or treated with 10 μM JSH-23 (dark grey). Period length of *Per2-dLuc* bioluminescence is shown (in hr), n = 6 subjects. (**D**) Cells were transfected with either siRNA against human *NF-κB p65* (pink) or non-targeting siRNA (Ctrl, blue). Period length of *Per2-dLuc* bioluminescence is shown (in hr), n = 7 subjects.

(**E**-**H**) Effects of *p65* overexpression in obese OAPs. (**E**) NF-κB activity in obese OAPs transfected with either 90 ng *p65* expression vector (*p65* OX, dark red) or empty backbone (Ctrl, red), n = 8 patients. (**F**) QRT-PCR measurement of *PER2* expression in non-synchronized OAPs obtained from obese patients. Cells were transfected with either the empty vector alone (Ctrl, red) or increasing doses of plasmid expressing *p65* vector (30-90 ng, *p65* overexpression, OX, gradient color to dark red) and/or of the empty backbone. Values are displayed as mRNA RA compared to values of Ctrl cells (n = 4 independent cultures/ dose, obtained from 2 patients). (**G**-**H**) Normalized bioluminescence of *Per2-dLuc* reporter oscillations in synchronized obese OAPs receiving 90 ng of *p65* expression vector (*p65* OX, dark red) or 90 ng of the empty backbone (Ctrl, red). (**G**) One representative trace per condition is shown. (**H**) Period length of *Per2-dLuc* bioluminescence in hr with n = 7 independent cultures/ dose, obtained from 3 obese patients.

(**I**) Normalized bioluminescence of *Per2-dLuc* reporter oscillations in synchronized non-obese OAPs obtained. Cells were transfected with either the empty vector alone (Ctrl, blue) or 90 ng of plasmid expressing *p65* vector (OX, indigo). Period length of *Per2-dLuc* bioluminescence is shown (in hr), with n = 7 subjects.

(**J**) ChIP analyses of p65 binding to the *CCL2* promoter. NF-κB p65 bound to *CCL2 κb sites 1, 2, 3* in the promoter (~200 bp, ~300 bp and 600 bp upstream to TSS) in OAPs isolated from non-obese (blue) or obese patients (red). Results are expressed in fold enrichment over IgG. Each histogram represents the mean ± SEM; n= 4 patients/ group.

(**K**-**M**) QRT-PCR measurement of *CCL2* expression in non-synchronized obese OAPs after targeted inhibition of NF-κB activity, either through (**K**) pharmacological or (**L**) gene silencing approaches, or after (**L**) NF-κB *p65* overexpression. (**K**) Cells were treated for 2hr with 10 μM NF-κB inhibitors Bay11-7085 (purple) or JSH-23 (grey) *vs*. vehicle (Ctrl, DMSO, red). Values are displayed as mRNA RA compared to values of untreated OAPs (n = 5 patients). (**L**) Cells were transfected with either siRNA against human *NF-κB p65* (violet) or non-targeting siRNA (Ctrl, red). Values are displayed as mRNA RA compared to values of Ctrl cells with n = 4 independent cultures per condition, obtained from 2 patients. (**M**) Cells were transfected with either the empty vector alone (Ctrl, red) or increasing doses of plasmid expressing *p65* vector (30-90 ng, *p65* overexpression, OX, gradient color to dark red) and/or of the empty backbone. Values are displayed as mRNA RA compared to values of Ctrl cells with n = 4 independent cultures/ dose, obtained from 2 patients.

Data are represented as mean ± SEM. *$p<0.05$, **$p<0.01$, ***$p<0.001$, paired two-tailed *t*-test (**B**-**E**, **H**, **I**, **L**), unpaired two-tailed *t*-test (**J**) or one-way repeated ANOVA followed by post hoc Dunnett's test (**A**, **F**, **K**, **M**). (**A**, **C**, **D**) not significant.

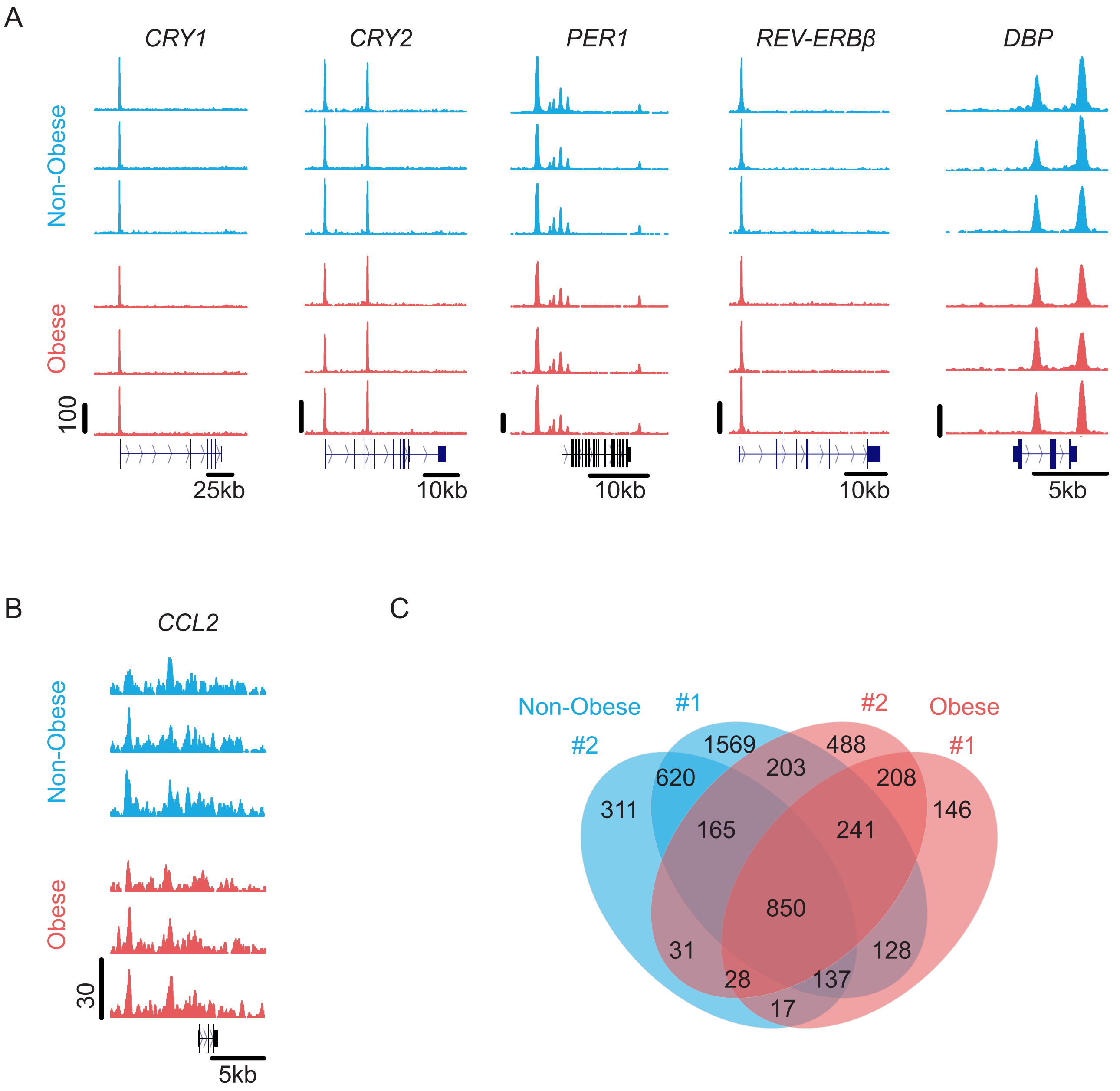

<u>Supplementary Figure 4. Occupancy of endogenous BMAL1 in OAPs: clock repressors and other targets</u> (related to Fig. 5-Fig. 8)

(**A**) UCSC genome browser images of BMAL1 ChIP-seq tracks at clock repressors in OAPs (n = 3 patients/ group). Normalized tag counts are indicated on the Y-axis and maximum track height is the same for all samples. The orientation for each gene is indicated below each browser track. (**B**) UCSC genome browser images of BMAL1 ChIP-seq tracks at *CCL2*. Normalized tag counts are indicated on the Y-axis and maximum track height is the same for all samples (n = 3 patients/ group). The orientation for each gene is indicated below each browser track. (**C**) VENN diagram depicting the detailed number of BMAL1 peaks and overlap in OAPs from 2 non-obese and 2 obese patients.

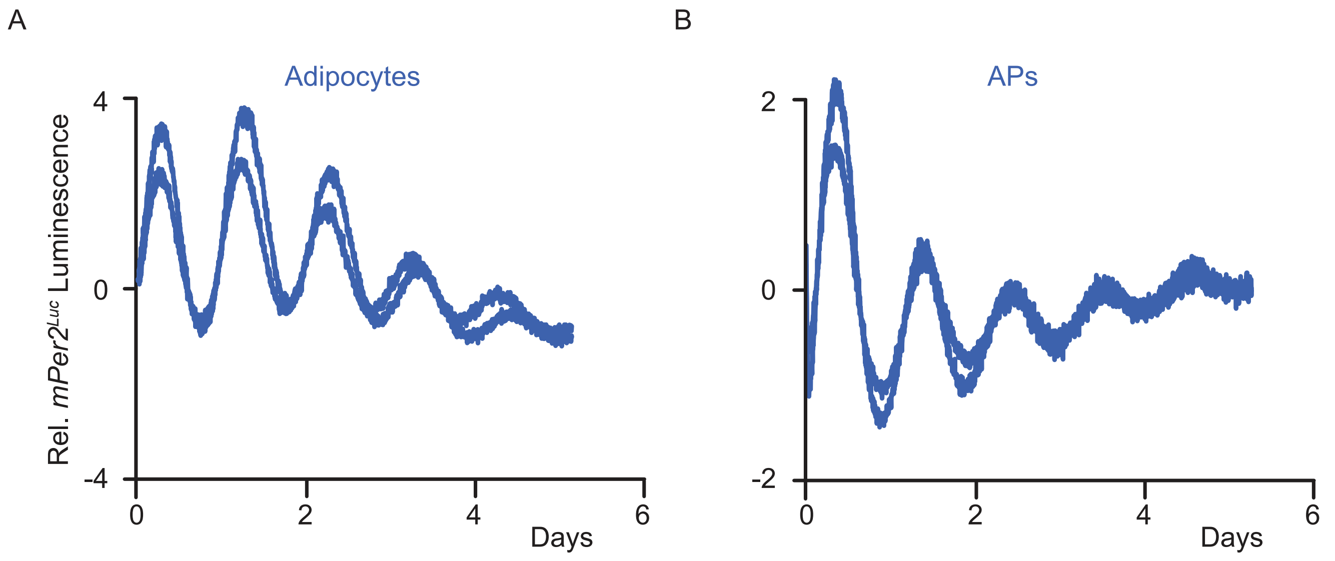

<u>Supplementary Figure 5. Adipocytes and adipocyte precursors display a cell-autonomous circadian clock in standard chow-fed mice</u> (related to Fig. 9)
Mice expressing the *Per2::Luciferase (mPer2^{Luc})* transcriptional reporter were used to collect epididymal AT. Adipocytes and adipocyte precursors were cultured separately to measure *ex vivo* PER2::LUCIFERASE fusion reporter protein from the endogenous *Per2* locus. (**A**-**B**) Normalized bioluminescence of reporter oscillations in epididymal mature adipocytes cultured in fibrin gels (**A**) and adipocyte precursors (AP, **B**). Two representative traces per condition are shown.

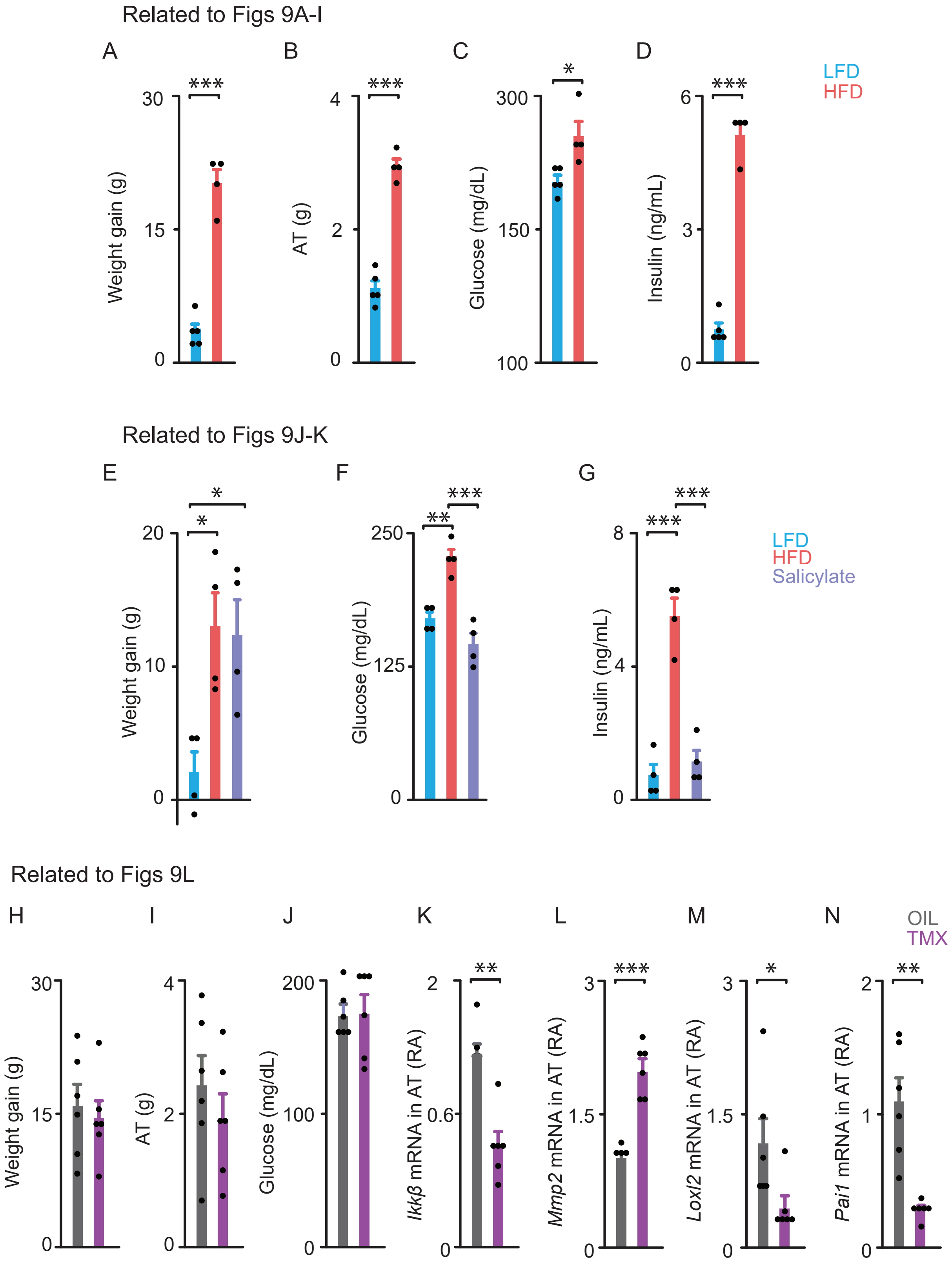

<u>Supplementary Figure 6. Metabolic parameters of the mouse cohorts</u> (related to Fig. 9)
(**A**, **E**, **H**) Body weight gain (g), (**B**, **I**) gonadal fat pad mass (g), (**C**, **F**, **J**), fed blood glucose (mg/dL) and (**D**, **G**) insulin (ng/mL) levels (at ZT8) in *mPer2^{Luc}* mice fed a LFD (blue), HFD (red), or HFD while receiving salicylate (purple). (**K-N**) QRT-PCR measurement of *Ikkβ, Mmp2, Loxl2, Pai1* expression in AT from HFD-fed Adipocyte *Ikkβ*-KO mice after oil (Ctrl, grey) or tamoxifen (dark purple) treatments. Data are represented as mean ± SEM, with (**A-D**) n = 5 LDF and n = 4 HFD-fed mice, (**E-G**) n = 4 mice per group or (**H-N**) n = 6 mice per group; *p<0.05, **p<0.01, ***p<0.001, by unpaired two-tailed *t*-test (**A-D**, **H-N**) or one-way ANOVA followed by post hoc Tukey’ s test (**E-G**). (**H-J**) not significant.

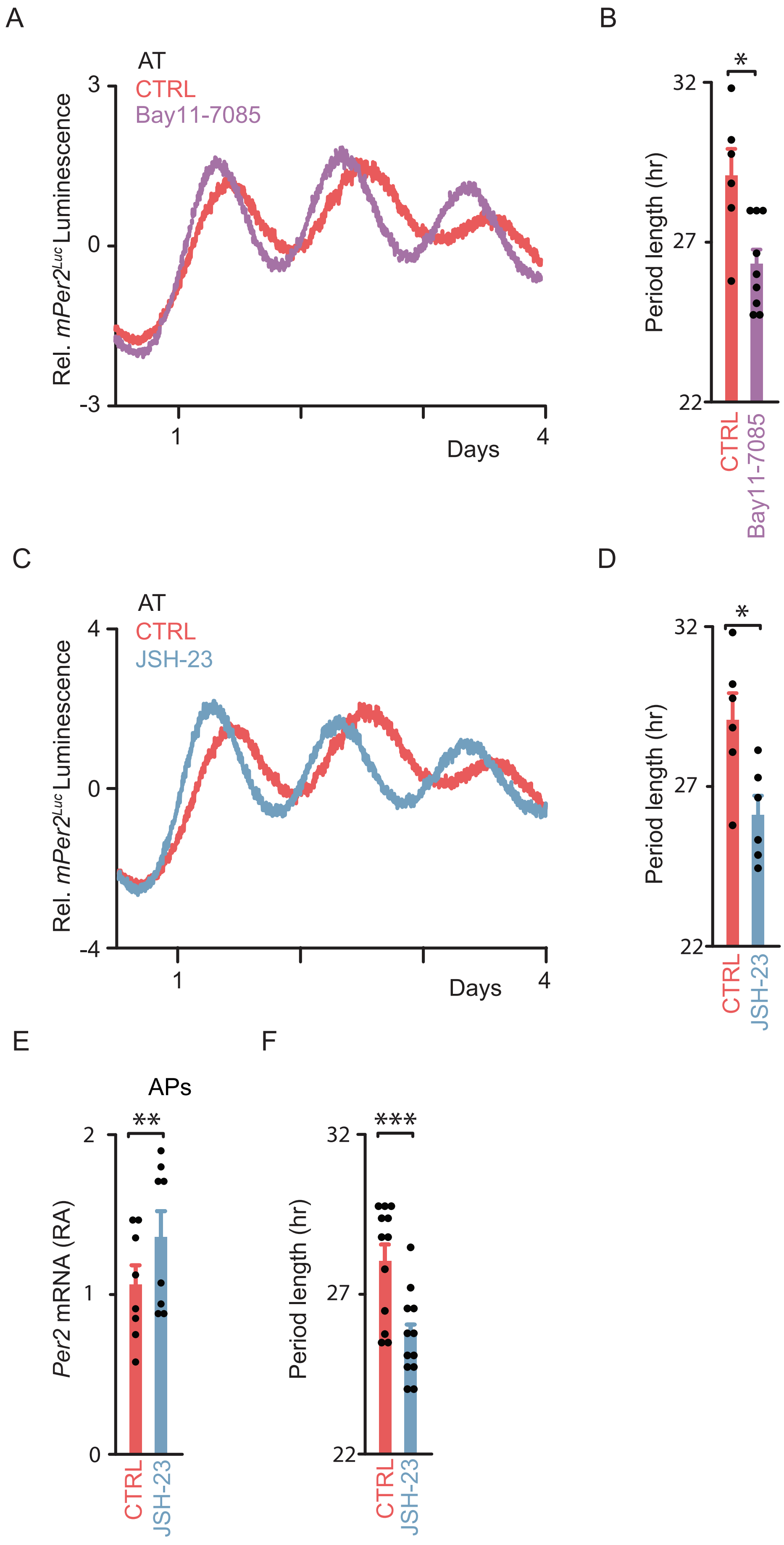

A
AT
CTRL
Bay11-7085
Rel. mPer2^Luc Luminescence
3
0
-3
1
Days
4
B
*
32
27
22
Period length (hr)
CTRL
Bay11-7085
C
AT
CTRL
JSH-23
Rel. mPer2^Luc Luminescence
4
0
-4
1
Days
4
D
*
32
27
22
Period length (hr)
CTRL
JSH-23
E
APs
**
2
1
0
Per2 mRNA (RA)
CTRL
JSH-23
F
***
32
27
22
Period length (hr)
CTRL
JSH-23

<u>Supplementary Figure 7. NF-κB inhibition shortens circadian period in adipose tissue from HFD-fed mice</u> (related to Fig. 9)

To further interrogate the involvement of NF-κB in the regulation of clock function, AT explants or APs obtained from HFD-fed mice (for 3 months) were treated with NF-κB inhibitors *ex vivo*. (**A**-**D**) Normalized bioluminescence of $mPer2^{Luc}$ reporter oscillations in AT. AT was isolated from HFD-fed $mPer2^{Luc}$ transgenic reporter line and explants were cultured with either 10 µM Bay11-7085 (purple), 10 µM JSH-23 (grey) or vehicle (CTRL, DMSO, red). (**A**, **C**) One representative trace per condition is shown (from n=6 DMSO and n=9 Bay11-7085 for **A**, from n=6 independent cultures/ conditions for **C**). (**B**, **D**) Period length of $mPer2^{Luc}$ bioluminescence in hr. Data are represented as mean ± SEM, with n=6 (DMSO) and n=9 (Bay11-7085) independent cultures per condition, from 3 HFD-fed mice (**B**) and with n=6 independent cultures per condition, from 3 HFD-fed mice (**D**). (**E**) QRT-PCR measurement of *Per2* gene expression in non-synchronized APs from HFD-fed mice following the addition of either DMSO (Ctrl, red) or JSH-23 (grey) to the culture medium for 2 hr. Data are represented as mean ± SEM, with n = 8 independent cultures per condition from 4 mice. (**F**) Normalized bioluminescence of $mPer2^{Luc}$ reporter oscillations in APs. APs were isolated from HFD-fed $mPer2^{Luc}$ mice and cultured with either 10 µM JSH-23 (grey) or vehicle (CTRL, DMSO, red). Period length of $mPer2^{Luc}$ bioluminescence is expressed in hr, Data are represented as mean ± SEM, with n = 12 independent cultures per condition from 3 mice.

*$p<0.05$, **$p<0.01$, ***$p<0.001$, paired two-tailed *t*-test.

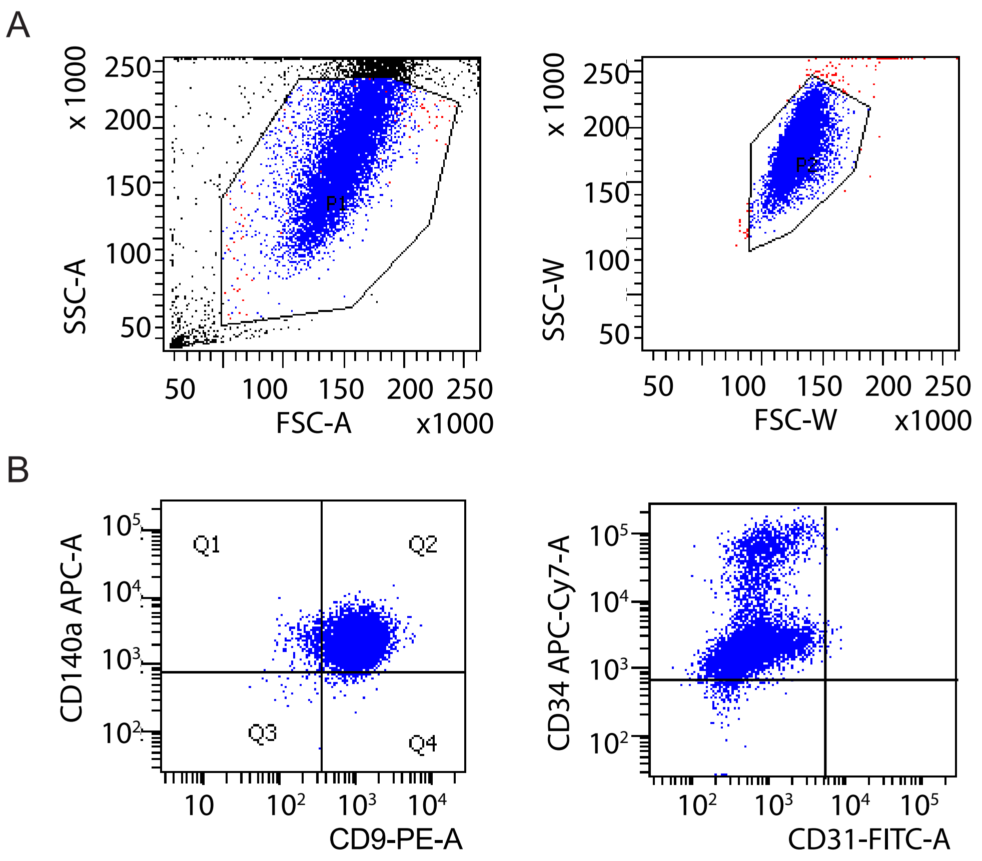

<u>Supplementary Figure 8. Gating strategy</u> (related to the Methods)

(**A**) The gate was used to discriminate the live cells (singlets) from the debris according to Side Scatter Light (SSC) and Forward Scatter Light (FSC)[5]. (**B**) Flow cytograms showing cells expressing PDGFα$^+$ (Alexa Fluor 647) and CD9 (PE), as shown in Fig 1 D-E. Detection of CD34 (APC-Cy7) in PDGFα$^+$ cells, with measurement of FITC intensity from conjugated anti-CD31 or anti-CD45 antibodies separately, as described in Methods (n=8 patients/group).

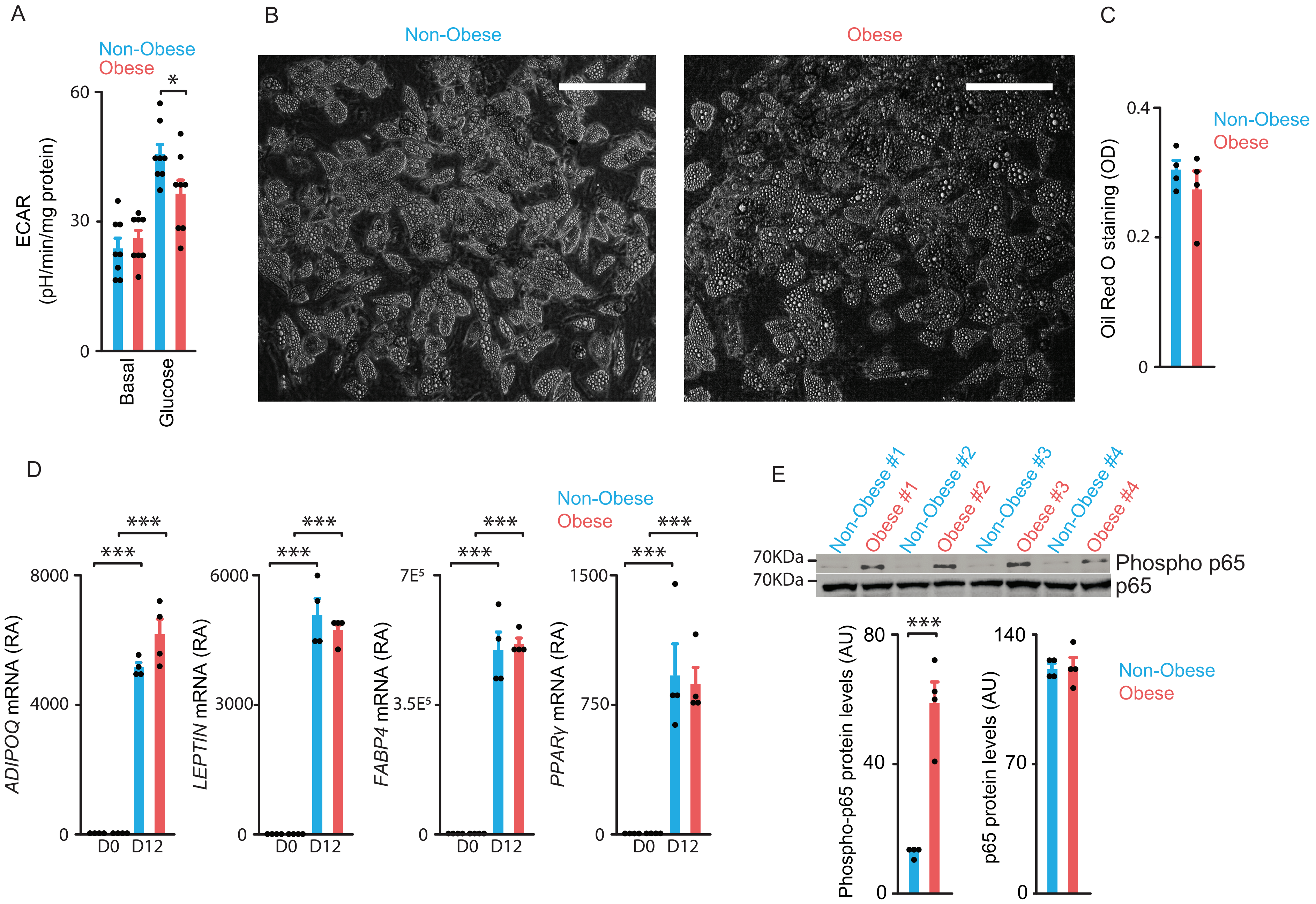

A
Non-Obese
Obese
ECAR (pH/min/mg protein)
60
30
0
*
Basal
Glucose
B
Non-Obese
Obese
C
Oil Red O staining (OD)
0.4
0.2
0
Non-Obese
Obese
D
Non-Obese
Obese
ADIPOQ mRNA (RA)
8000
4000
0
D0
D12
***
***
LEPTIN mRNA (RA)
6000
3000
0
D0
D12
***
***
FABP4 mRNA (RA)
7E5
3.5E5
0
D0
D12
***
***
PPARγ mRNA (RA)
1500
750
0
D0
D12
***
***
E
Non-Obese #1
Obese #1
Non-Obese #2
Obese #2
Non-Obese #3
Obese #3
Non-Obese #4
Obese #4
70KDa
70KDa
Phospho p65
p65
Phospho-p65 protein levels (AU)
80
40
0
***
p65 protein levels (AU)
140
70
0
Non-Obese
Obese

Supplementary Figure 9. Characterization of OAPs (PDGFRα$^+$ CD34$^+$ CD31$^-$ CD45$^-$) (related to the Methods)

(**A**) ECAR from non-obese (blue) and obese (red) OAPs. Cells are either untreated (basal) or treated with glucose 10 mM. ECAR was similar between obese and non-obese OAPs in the basal state (not significantly different), while glucose-induced ECAR was slightly decreased in obese OAPs.

(**B**-**D**) Measurement of *in vitro* adipogenesis. (**B**) Confluent OAPs from non-obese and obese patients after treatment with an adipogenic medium (at day 12, D12). The differentiation of OAPs into lipid-filled cells was assessed morphologically. Cells acquired a typical round shape, and at day 12, more than 70% of them had accumulated lipid droplets. The scale bar represents 100 µm. Representative pictures from n=4/ group. (**C**) Quantification of Oil Red O staining (at D12) from non-obese (blue) and obese (red) patients. Values are displayed in OD. No significant difference was found. (**D**) Gene expression of adipogenic markers (at day 0 and 12, D0, D12). Values are displayed as mRNA RA.

(**E**) Western blot analysis of total NF-κB p65 and phospho p65, provided as an additional control for the measurement of NF-κB activity (Fig. 1F). Total NF-κB p65 and phospho p65 were measured in OAPs from 4 non-obese (blue) and 4 obese (red) patients. Data from the Western Blot (*top*) were quantified using Fiji (intensity, Arbitrary Units). Non-obese and obese OAPs displayed similar total p65 levels (not significantly different), while phospho p65 was increased in obese OAPs (molecular weight < 70 KDa).

All histograms above are represented as mean ± SEM, with n = 8 patients/ group (and average of 6 replicates for each patient) in **A** or n = 4 patients/ group in **C-E**. *p<0.05, **p<0.01, ***p<0.001, by unpaired two-tailed t-test (**C**, **E**); or unpaired (**A**) and paired (**D**) differences identified two-way ANOVA followed by post hoc Sidak' s test. (**C**) No significant difference was found.

Glucose significantly induced ECAR levels in both non-obese and obese OAPs (***p<0.001) (**A)**; there was a significant effect of obesity on *ADIPOQ* mRNA at D12(*p<0.05) (**D**), not indicated.

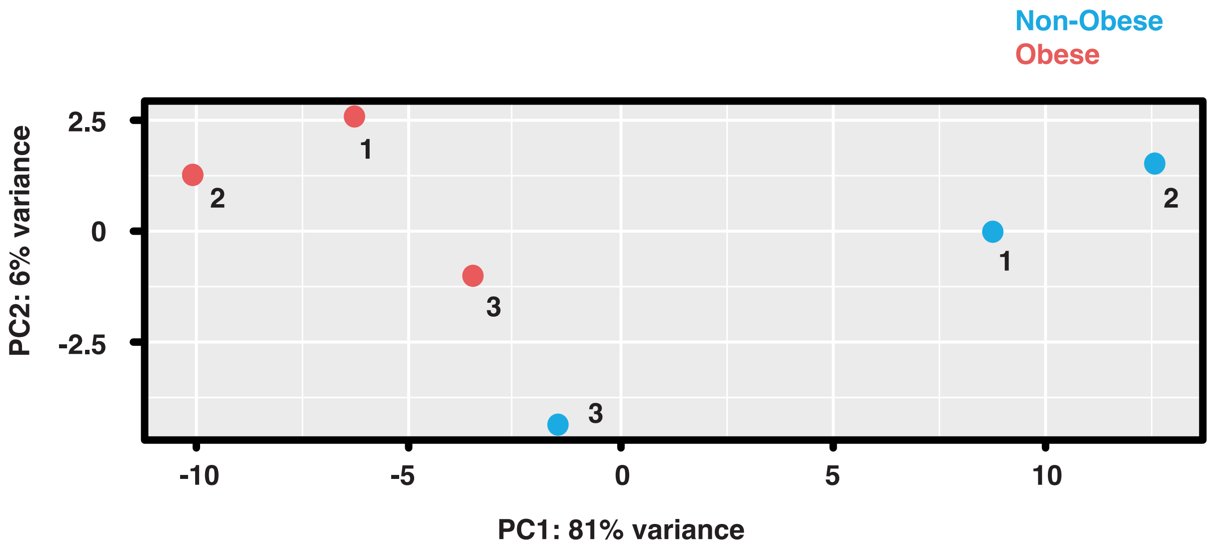

<u>Supplementary Figure 10. Dispersion between the ChIP-seq reads in the BMAL1 peak regions in human omental adipocyte precursors</u> (related to the Methods)

Principal components analysis (PCA) plot of samples. Dispersion between the reads in the BMAL1 peak regions in OAPs from 3 non-obese (blue) and 3 obese (red) patients has been determined by DESeq2 analysis[6]. Patient number (1, 2, 3) is indicated for each condition. The PCA plot suggests that the condition non-obese/obese contributes to establishing the BMAL1 cistrome of OAPs.

Genes	Distance to TSS	Sequences
PER2 (NM_022817)	-210<br>-928	gccggaagTTCCttg<br>cgcggactTTCCcgc
CRY1 (NM_004075)	-1070<br>-1185	ctcggcgaTTCCtcc<br>gtGGGAggctccacg
REV-ERBα (NM_021724)	-1032<br>-1183	agggagatTTCCctg<br>acaGGGAgtccctac
RORα (NM_134261)	-1190	atgGGGAaacccaca
BMAL1 (NM_001297724)	-990<br>-1073<br>-1228	gaGGGAcatcccggg<br>tccgggcaTTCCgac<br>gaaggcatTTCCacc
CLOCK (NM_004898)	-794<br>-1092	gatggaggTTCCagt<br>aagggcatTTCCtga
DBP (NM_001352)	-1073	agGGGAgttacccct

Supplementary Table 1. Human core clock genes possess NF-κB binding sites

*S*equence analysis using the promoter prediction and regulatory sequence algorithm (http://www.genomatix.de/matinspector/)[7] identified putative NF-κB binding sites to the promoter regions of human core clock genes. TSS is defined here as the first ATG codon (methionine). This table is not exhaustive, and only describes the sites located up to 1800bp upstream to the TSS.

	Cohort 2	Cohort 3
Age (years)	36.3 ± 2.6	57.7 ± 2.6
Number (Sex ratio Men/Women)	12 (6/6)	3 (1/2)
BMI (kg/m^2)	45.8 ± 1.6	39.5 ± 2.4
Systolic blood pressure (cmHg)	14.6 ± 0.5	14.5 ± 0.9
Diastolic blood pressure (cmHg)	9.3 ± 0.4	8.9 ± 0.2
Fasting glucose (mmol/l)	6.1 ± 0.4	6.2 ± 0.5
HDL-cholesterol (mmol/l))	1 ± 0.1	N/A
Total cholesterol/HDL	3.6 ± 0.2	N/A
Triglycerides (mmol/l)	1.3 ± 0.1	N/A

Supplementary Table 2. Clinical and laboratory characteristics of obese patients

Clinical and laboratory parameters of these additional patients were measured after an overnight fast before surgery. Paired biopsies of omental *vs.* subcutaneous adipose tissue have been obtained from the cohort 2 (Supplementary Fig.1D-E). Culture and treatment of mature adipocytes *in vitro* has been performed on cells isolated from the cohort 3 (Fig. 3H). Data are presented as mean ± SEM with n = 12 patients (cohort 2) or n = 3 patients (cohort 3).

A

Genes	Sequences
ADIPOQ	TGAAGGATGTGAAGGTCAGCC<br>TCCCCATACACCTGGAGCC
BMAL1	CATTAAGAGGTGCCACCAATCC<br>CAAAAATCCATCTGCTGCCC
CCL2	TCAGCCAGATGCAATCAATG<br>GCACTGAGATCTTCCTATTGGTG
CCL4	GCTGCTTTTCTTACACCGCG<br>GGTTTGGAATACCACAGCTGG
CCL5	AGCCCTCGCTGTCATCCTC<br>GGGCAATGTAGGCAAAGCAG
CD68	ACAATGTGTCCTTCCCCCAC<br>GCTGCAACTGAAGCTCTGCC
CLOCK	CTGTAGCTTGTGGGGCAGTC<br>AGGATATGCAGTCACCACCTG
CRY1	TGACACAGCTTCGTCAGGAG<br>TGTCAGGAAGCAAGCAACTG
DBP	GAAAAATCCAGGTGCCGGA<br>CGTTGTTCTTGTACCGCCG
FABP4	CATGTGCAGAAATGGGATGG<br>CTCGTGGAAGTGACGCCTTT
FLOT1	ATTGCCCTGGAGACGTTAGA<br>AGGTCTGAGGAGGCCACTTT
GADPH	TGTTCGTCATGGGTGTGAAC<br>CTAAGCAGTTGGTGGTGCAG
GPX1	TGCGGGGCAAGGTACTACTTA<br>AGCTCGTTCATCTGGGTGTAG
IL6	AGCCAGAGCTGTGCAGATGA<br>CATTTGTGGTTGGGTCAGGG
LEPTIN	AAGCTGTGCCCATCCAAAAA<br>GGAGGAGACTGACTGCGTGTG
LOXL2	CTCCTACGGCAAGGGAGAAG<br>TCGTTGCCAGTACAGTGGAG
MLL1	AGCGGAGAGGATGAGCAAT<br>GGGACTTCGCACTCTGACTT
MMP2	GAAGGATGGCAAGTACGGCT<br>GGAATGGAAACTTGCAGGGC
NCOR2	CCTTTCCTACCCAGTGCAGA<br>GACAGGTGGGAGGCATAGTC
P50	GTGAGGATGGGATCTGCACT<br>GAAGCCGACCACCATGTC
P65	GGCGAGAGGAGCACAGATAC<br>CCTGGTCCTGTGTAGCCATT
PER2	TGGAAGCATTGACTCCTCAG<br>CTGCAGGACGCACTTAATGA
PPARγ	GGCCAAGGCTTCATGACAAG<br>AAAAGGCTTTCGCAGGCTCT
PAI1	CCCATGATGGCTCAGACCAA<br>GGCAGTTCCAGGATGTCGTA
REV-ERBα	GAAGCGACATTGCTGGCAG<br>GTCGGAGCATCCAGCAGAAC
RORα	AGCAATGCCACCTACTCCTG<br>CAGCGGTTTCTACTGGTTCG
TNFα	CTCTTCTGCCTGCTGCACTTT<br>GATGATCTGACTGCCTGGGC

B

Genes	Sequences
CCL2 Ebox	CCCTGCTTCCCTTTCCTACT<br>CTTATTGAAAGCGGGCAGAG
CCL2 kB1	CCCATTTGCTCATTTGGTCT<br>ATGCAGCATCCTGTGGATTT
CCL2 kB2	CCTCGCTTCCCTTTTCT<br>CTGCTGAGACCAAATGAGCA
CCL2 kB3	TGCCTTTGTCCAAGTCTGAA<br>GCTCCCTGACAATGCATTAAA
FLOT1 Ebox	CCCAATTTTCAGGAGCACAT<br>CAGCTGCGAGAGTGACACAT
GPX1 Ebox	CCAGCCCTTGGAAGGGTAAC<br>AGGAAAAGGCTGGAGAGTGC
LOXL2 Ebox	CAGGGGAAAGGATCTGTGAA<br>TCATGCCCAGAAGGAAAGTT
MLL1 Ebox	CATCCTCCGCGTAGTCCTC<br>CGCAACCTGGATAACTGCAT
MMP2 Ebox	CAAGTACGCACAACACGCAC<br>AGCAGGGGCTGAGTAACAAC
NCOR2 Ebox	TCAGAGGGTTCTGTCGAGTG<br>GGAGCCTCGGGATTAAAAAC
PAI1 Ebox	CGTGTATCATCGGAGGCGG<br>GCCAGCCACGTGATTGTCT
PER2 κBsite	GGACTCCTCGGCTTGAAAC<br>GCTCGACCACTCTCCACCT
PER2 Ebox	GCGGTCACGTTTTCCACTAT<br>AGCTGCACGTATCCCCTCAG
PER2 E19	CCGGAATAGATTCACCTTGG<br>CCGCTTGGACTTCAATTTTC
PER2 3'UTR	AGAGCTTTCCCAGGGTGTTT<br>CAGTCCCCAAGAGAGGACAA

C

Genes	Sequences
18S	CGGCTACCACATCCAAGGAA<br>GGGCCTCGAAAGAGTCCTGT
Ccl2	CAGCAGGTGTCCCAAAGAAG<br>CCTCTCTCTTGAGCTTGGTGA
Ccl4	TTCTGTGCTCCAGGGTTCTC<br>AGGAAGTGGGAGGGTCAGAG
Ccl5	TGCCCACGTCAAGGAGTATTT<br>TCTCTGGGTTGGCACACACTT
Dbp	GCAGAGTCCTGTTCCTTGCT<br>CTGCATCATGACGTTCTTCG
F4/80	TGCATAATCGCTGCTGGTTG<br>CCAGGCAAGGAGGACAGAGTT
Gadph	CAAGGAGTAAGAAACCCTGGACC<br>CGAGTTGGGATAGGGCCTCT
Ikkβ	CAGCCCAAAGAACAGAGACC<br>ACCACATTGGGATGGTTCAG
Il6	TTCCATCCAGTTGCCTTCTTG<br>TTGGGAGTGGTATCCTCTGTG
Loxl2	AGCTGTGGTCAGTTGTGTGC<br>AAGGGTTGCTCTGGCTTGTA
Mmp2	ACCCAGATGTGGCCAACTAC<br>AAAGCATCATCCACGGTTTC
Pai1	CGGCAGATCCAAGATGCTAT<br>GGCATCCGCAGTACTGATCT
Per2	TGTGCGATGATGATTCGTGA<br>GGTGAAGGTACGTTTGGTTTGC
Rev-erbα	ATGCCCATGACAAGTTAGGC<br>GGGCTACCTGATGCATGATT

Supplementary Table 3. Gene sequences used as primers for quantitative real-time PCR

Primer sequences used in QRT-PCR assays in human (**A**-**B**) or mouse (**C**) tissues. Primers were used for either (**A**, **C**) analysis of expression following RNA extraction or **(B)** for determination of NF-κB p65, BMAL1 or RNA POLII binding following conventional ChIP.

Supplementary References

1 Robinson, J. T. *et al.* Integrative genomics viewer. *Nat Biotechnol* **29**, 24-26, doi:10.1038/nbt.1754 (2011).

2 Thorvaldsdottir, H., Robinson, J. T. & Mesirov, J. P. Integrative Genomics Viewer (IGV): high-performance genomics data visualization and exploration. *Brief Bioinform* **14**, 178-192, doi:10.1093/bib/bbs017 (2013).

3 Kasowski, M. *et al.* Variation in transcription factor binding among humans. *Science* **328**, 232-235, doi:10.1126/science.1183621 (2010).

4 Wu, Y. *et al.* Reciprocal Regulation between the Circadian Clock and Hypoxia Signaling at the Genome Level in Mammals. *Cell Metab* **25**, 73-85, doi:10.1016/j.cmet.2016.09.009 (2017).

5 Brusa, D and Balligand,J.L., Classification of the Immune Composition in the Tumor Infiltrate Methods Mol Biol2019;1979:305-315. doi: 10.1007/978-1-4939-9240-9_19.

6 Love, M.I., Huber, W. & Anders, S. Moderated estimation of fold change and dispersion for RNA-seq data with DESeq2. *Genome Biol* **15**(12):550, doi:10.1186/s13059-014-0550-8 (2014)

7 Cartharius K*, et al.* MatInspector and beyond: promoter analysis based on transcription factor binding sites. *Bioinformatics* **21**, 2933-2942 (2005).
