# Supplementary material for: Circadian clock dysfunction in human omental fat links obesity to metabolic inflammation
Source: Nat Commun. 2021 Apr 22;12:2388. doi: 10.1038/s41467-021-22571-9 (PMC8062496; doi:10.1038/s41467-021-22571-9)
Supplement: Supplementary file 3 — Reporting Summary [file 41467_2021_22571_MOESM3_ESM.pdf]

nature research

Corresponding author(s): Eleonore Maury

Last updated by author(s): Jan 28, 2021

Reporting Summary

Nature Research wishes to improve the reproducibility of the work that we publish. This form provides structure for consistency and transparency in reporting. For further information on Nature Research policies, see Authors & Referees and the Editorial Policy Checklist .

Statistics

For all statistical analyses, confirm that the following items are present in the figure legend, table legend, main text, or Methods section.

n/a	Confirmed	
☐	☒	The exact sample size (*n*) for each experimental group/condition, given as a discrete number and unit of measurement
☐	☒	A statement on whether measurements were taken from distinct samples or whether the same sample was measured repeatedly
☐	☒	The statistical test(s) used AND whether they are one- or two-sided<br>*Only common tests should be described solely by name; describe more complex techniques in the Methods section.*
☐	☒	A description of all covariates tested
☐	☒	A description of any assumptions or corrections, such as tests of normality and adjustment for multiple comparisons
☐	☒	A full description of the statistical parameters including central tendency (e.g. means) or other basic estimates (e.g. regression coefficient) AND variation (e.g. standard deviation) or associated estimates of uncertainty (e.g. confidence intervals)
☐	☒	For null hypothesis testing, the test statistic (e.g. *F*, *t*, *r*) with confidence intervals, effect sizes, degrees of freedom and *P* value noted<br>*Give P values as exact values whenever suitable.*
☒	☐	For Bayesian analysis, information on the choice of priors and Markov chain Monte Carlo settings
☒	☐	For hierarchical and complex designs, identification of the appropriate level for tests and full reporting of outcomes
☒	☐	Estimates of effect sizes (e.g. Cohen's *d*, Pearson's *r*), indicating how they were calculated

Our web collection on statistics for biologists contains articles on many of the points above.

Software and code

Policy information about availability of computer code

Data collection	Kronos Dio software (Atto Kronos v2)<br>BD FACSDiva software v8
Data analysis	-Prism 8 GraphPad software<br>-FlowJo software v10<br>-Fiji https://imagej.net/Fiji with ImageJ v2<br>-Metacycle v1.2 https://cran.r-project.org/web/packages/MetaCycle/index.html<br>was used with the custom script deposited in Github:<br>https://github.com/EmauryUCL/Extraction-Analysis-and-Graph-of-Lumicycle-data/releases/tag/V1.0<br>-UCSC Genome browser http://genome.ucsc.edu/index.htlm<br>-Integrative genome viewer http://www.broadinstitute.org/igv/<br>-Genomatix Matinspector v8.4 https://www.genomatix.de/matinspector/<br>–MACS v2.1.0 algorithm<br>(Zhang Y, et al. Model-based analysis of ChIP-Seq (MACS). Genome Biol 9, R137 (2008)).<br>-DESeq2 analysis software package (v1.24.0) (Love MI, Huber W, Anders S. Moderated estimation of fold change and dispersion for RNA-seq data with DESeq2. Genome Biol 15, 550 (2014))<br>-HOMER v4 http://homer.ucsd.edu/homer/index.html Heinz S, et al. Simple combinations of lineage-determining transcription factors prime cis- regulatory elements required for macrophage and B cell identities. Mol Cell 38, 576-589 (2010).<br>-KEGG pathway database: The analysis was performed using clusterProfiler https://www.genome.jp/kegg/. (https://bioconductor.org/packages/release/bioc/html/clusterProfiler.html)<br>Relevant literature: 10.1089/omi.2011.0118<br>-Gene ontology analysis was also performed with clusterProfiler, using the “Biological Process” sub-ontology<br>The analysis used Bioconductor’s human annotation database (http://bioconductor.org/packages/release/data/annotation/html/org.Hs.eg.db.html).

-The code for the Burrows-Wheeler alignment algorithm can be downloaded from http://bio-bwa.sourceforge.net/
Relevant literature: 10.1093/bioinformatics/btp324

For manuscripts utilizing custom algorithms or software that are central to the research but not yet described in published literature, software must be made available to editors/reviewers. We strongly encourage code deposition in a community repository (e.g. GitHub). See the Nature Research guidelines for submitting code & software for further information.

Data

Policy information about availability of data

All manuscripts must include a data availability statement. This statement should provide the following information, where applicable:

- Accession codes, unique identifiers, or web links for publicly available datasets
- A list of figures that have associated raw data
- A description of any restrictions on data availability

Data availability:
ChIP-seq data generated and analyzed during this study are available in
a GEO repository with the identifier GSE149064.
(publicly available, https://www.ncbi.nlm.nih.gov/geo/query/acc.cgi?acc=GSE149064).
We used the publicly available data sets GSE19486 and GSE85096:
https://www.ncbi.nlm.nih.gov/geo/query/acc.cgi?acc=GSE19486
DOI: 10.1126/science.1183621
https://www.ncbi.nlm.nih.gov/geo/query/acc.cgi?acc=GSE85096
DOI: 10.1016/j.cmet.2016.09.009
The other data sets generated and analyzed during the study are provided in Data Source.

Code availability:
The analysis of bioluminescence data was performed with a custom script deposited in Github: https://github.com/EmauryUCL/Extraction-Analysis-and-Graph-of-Lumicycle-data/releases/tag/V1.0
The analysis of KEGG pathway database was performed using clusterProfiler https://www.genome.jp/kegg/.(https://bioconductor.org/packages/release/bioc/html/clusterProfiler.html). The gene ontology analysis was also performed with clusterProfiler, using the “Biological Process” sub-ontology, using Bioconductor’s human annotation database (http://bioconductor.org/packages/release/data/annotation/html/org.Hs.eg.db.html).
The code for the Burrows-Wheeler alignment algorithm can be downloaded from:
http://bio-bwa.sourceforge.net/

Field-specific reporting

Please select the one below that is the best fit for your research. If you are not sure, read the appropriate sections before making your selection.

[x] Life sciences [ ] Behavioural & social sciences [ ] Ecological, evolutionary & environmental sciences

For a reference copy of the document with all sections, see nature.com/documents/nr-reporting-summary-flat.pdf

Life sciences study design

All studies must disclose on these points even when the disclosure is negative.

Sample size

-Human Cohort 1;
For the expression of cytokines and chemokines in human omental adipose tissue from non-obese and obese patients:
Needed sample sizes were initially estimated based on our previous works (Maury E, et al., 2007), indicating N=5 per group would be needed for power of 0.80 and alpha of 0.05. Thus, a first set of experiments has been initially performed with N=5 patients per group. Using this original cohort, significant differences in chemokine gene expression were found between the two groups. Then, the cohorts of patients have been increased (N=8 patients per group, see Table 1). Using this larger cohort, we were able to validate and strengthen our human data. To further validate our results, additional cohorts have also been included (see Table S2).
-Human subjects, Cohort 2:
Differential expression of chemokines in paired subcutaneous vs. visceral adipose tissue biopsies. We determined the size of cohort 2 based on previous works describing paired comparison of chemokine gene expression in subcutaneous vs. visceral tissue. For example, the highly cited article from Bruun et al., 2005 “Monocyte Chemoattractant Protein-1 Release Is Higher in Visceral than Subcutaneous Human Adipose Tissue (AT): Implication of Macrophages Resident in the AT”, states that visceral fat displayed a 2-fold increase in CCL2 gene expression (and CCL2 secretion) compared with subcutaneous fat from both lean and obese subjects. These prior data indicated that we would need to study n=10 subjects (10 paired subcutaneous and visceral adipose tissues) to be able to reject the null hypothesis that this response difference is zero with power of 0,8 and with alpha of 0,05. For the experimental design, we increased this number by 20% in order to strengthen the data (n=12 subjects/ group).
-Human subjects, Cohort 3:
We tested the hypothesis that a potent NF-kB inhibitor regulates PER2 expression in freshly isolated mature adipocytes, as found in OAPs. We determined the size of cohort 3 based on our data obtained in cohort 1, showing that NF-kB induces a strong increase in PER2

expression in OAPs (6-fold increase or more). We determined that we would need to study n= 2 subjects to be able to reject the null hypothesis that this response difference is zero with power 0,8 and with alpha of 0,05. For the experimental design, we increased this number to n=3 subjects/group in order to strengthen the data.

-Mice:
The data from Hong et al., 2018 “Requirement for NF-kB in maintenance of molecular and behavioral circadian rhythms in mice” has been used to determine that n=4 mice per group would be required to observe a significant effect of HFD on the expression of PERIOD genes in epididymal adipose tissue (with HFD, 60%Kcal vs. 45%Kcal from fat in the prior study). Thus, for a difference in the experimental (HFD) and control (LFD) means of 2.5, we would need n=4 mice per group to be able to reject the null hypothesis that the population means of the experimental and control groups are equal with power of 0,8 and with alpha of 0,05. This number has been increased when tamoxifen treatment was required (n=6 mice per group) to anticipate an eventual loss of mice or data exclusion, which did not occur.

Data exclusions

Exclusion criteria were pre-established: any exclusion decision was supported by the use of the Grubbs test for outlier detection.
-Human subjects:
No data were excluded from the study, except for Fig S1E where 2 significant outliers have been found using Grubbs test as described in Fig S1E legend. Specifically, when analyzing clock gene expression in paired subcutaneous vs. omental adipose tissue biopsies from 12 patients, the Grubbs’ test identified one outlier for expression of CRY1 in omental adipose tissue (i.e., in one of the 12 patients), thereby leading to the exclusion of this value from CRY1 expression analysis. Similarly, the Grubbs’ test identified one outlier for expression of ROR in omental adipose tissue (i.e., in one of the 12 patients), thereby leading to the exclusion of this value from ROR expression analysis.
-Experiments performed in mice: All tissues were carefully examined during necropsy and sampling (to detect any lesions). No mouse was excluded and no outlier has been identified.

Replication

A first set of experiments has been initially performed with N=5 patients per group. Then, the cohorts of patients have been increased (N=8 patients per group). Using this larger cohort, we were able to validate and strengthen our human data. There is no further replication: all the experiments, which were performed for this study, are shown in the manuscript.

Randomization

Patients were age- and sex-matched (obese patients (class II and III; Body Mass Index BMI >35 kg/m2; 51.8+/- 3.9 yrs) and non-obese patients (BMI <30 kg/m2, with either normal weight or slightly overweight; 50.1+/-3.4 yrs).
When mice were used, groups were age- and sex-matched. Cages were randomly assigned to experimental groups to ensure that each group was matched for body weight at the beginning of the feeding experiment.

Blinding

No blinding procedure was used: although de-identified, the authors knew when the subject was an obese or non-obese patient. Actually, to limit the impact of inter-assays variations (when using luminometer or flow cytometry devices), experiments included the same number of non-obese and obese patients per group (studied simultaneously). However, donors in each group were chosen at random to avoid any bias of selection.

Reporting for specific materials, systems and methods

We require information from authors about some types of materials, experimental systems and methods used in many studies. Here, indicate whether each material, system or method listed is relevant to your study. If you are not sure if a list item applies to your research, read the appropriate section before selecting a response.

Materials & experimental systems

n/a	Involved in the study	
	x	Antibodies
x		Eukaryotic cell lines
x		Palaeontology
	x	Animals and other organisms
	x	Human research participants
x		Clinical data

Methods

n/a	Involved in the study	
	x	ChIP-seq
	x	Flow cytometry
x		MRI-based neuroimaging

Antibodies

Antibodies used

BMAL1 antibody Abcam Catalog No: Ab3350 (lot GR297717-5);
RNA polymerase II CTD repeat YSPTSPS phospho S5 antibody Abcam Catalog No: Ab5131 (lot GR3178102-1);
NFκB p65 antibody Active Motif Catalog No: 39369 (lot 27308001);
Anti-rabbit IgG, HRP-linked Antibody Cell Signaling Technology Catalog No: 7074 (lot24);
Alexa Fluor® 647 mouse anti-human CD140a BD Biosciences Catalog No: 562798 (clone alphaR1, lot 7132680);
PE Mouse anti-human CD9 BD Biosciences Catalog No: 555372 (clone M-L13, lot 7268691);
mouse anti-human CD45-FITC Immunotools Catalog No: 21810453X2 (lot Sp034);

mouse anti-human CD31-FITC Miltenyi Biotec Catalog No: 130-117-539
rabbit anti-human phospho S536 Abcam Catalog No : Ab86299
APC/Cyanine7 mouse anti-human CD34 BioLegend Catalog No: 343613
(Normal Rabbit Immunoglobulin G, Preprotech Catalog No: 500-P00)
All the names and catalog numbers can be found in the Methods.

Validation

-Conventional ChIP assays:
All antibodies are commercially available and were previously validated by the manufacturer for the required purposes. Besides these validations, an extensive list of publications is available (e.g. for BMAL1 ChIP: Ab3350, see Wu Y et al, Cell Metab. 2017 Jan 10;25(1):73-85.; for p65 ChIP: Raaz U et al, Circ Res 2015 Aug 28; 117(6): 513-524; for RNA polymerase II CTDrepeat YSPTSPS Ser5P, ab5131 has been references in more than 275 publications).
Specifically Anti- RNA POLII CTD repeat YSPTSPS Ser-5P antibody (Abcam, #Ab5131), https://www.abcam.com/rna-polymerase-ii-ctd-repeat-ysptsps-phospho-s5-antibody-ab5131.html https://www.abcam.com/rna-polymerase-ii-ctd-repeat-ysptsps-phospho-s5-antibody-ab5131/reviews/45979?productWallTab=ShowAll . The antibody was diluted at 1:250. The antibody had already been validated for this application using human cells: Chen YJ et al. SMYD3 Promotes Homologous Recombination via Regulation of H3K4-mediated Gene Expression. Sci Rep 7:3842 (2017). Additional references: Liu X et al. Positive feedback loop mediated by protein phosphatase 1a mobilization of P-TEFb and basal CDK1 drives androgen receptor in prostate cancer. Nucleic Acids Res 45:3738-3751 (2017). Ding N et al. BRD4 is a novel therapeutic target for liver fibrosis. Proc Natl Acad Sci U S A 112:15713-8 (2015). The dilution suggested by the manufacturer is 1:100. Running both conditions simultaneously for the initial experiment, the results were qualitatively similar with a 1:250 dilution.
The antibodies against p65 (Active Motif, #39369) and BMAL1 (abcam#Ab3350) were diluted at 1:250.
-Western-blots:
anti-p65 at 1:5000 (https://www.activemotif.com/catalog/details/39369/nfkb-p65-antibody-pab-3), phopsho p65 at 1:2000 (https://www.abcam.com/nf-kb-p65-phospho-s536-antibody-ab86299.html) and the secondary antibody at 1:2000 (https://www.cellsignal.com/products/secondary-antibodies/anti-rabbit-igg-hrp-linked-antibody/7074). These dilutions are already indicated on the manufacturer's website.
-Flow cytometry:
The antibodies were already validated for this application using human cells. Thus, we used the concentrations provided by the manufacturers, i.e, 1 × 10^6 cells are stained in a 100-µl experimental sample, including the following: 5ul anti-PDGFRa antibody.
(https://www.bdbiosciences.com/eu/applications/research/stem-cell-research/ectoderm-markers/human/alexa-fluor-647-mouse-anti-human-cd140a-r1-also-known-as-alpha-r1/p/562798)
20ul anti-CD9 (https://www.bdbiosciences.com/eu/applications/research/stem-cell-research/pluripotent-stem-cell-markers-esc-and-ipsc/human/pe-mouse-anti-human-cd9-m-l13/p/555372)
5 µl anti-CD34 (https://www.biolegend.com/en-us/products/apc-cyanine7-anti-human-cd34-antibody-12973)
and with 5 µl anti-C45 http://www.immunotools.de/html/datas-fitc/21810453.pdf or
2 µl anti-CD31 https://www.miltenyibiotec.com/BE-en/products/cd31-antibody-anti-human-ac128.html#gref https://www.miltenyibiotec.com/BE-en/applications/all-protocols/cell-surface-flow-cytometry-staining-protocol-pbs-edta-bsa-1-50.html . Preliminary experiments aimed at testing the antibodies were performed using a FACScalibur flow cytometer, and Cellquest v5, using the concentrations provided by the manufacturers.
-ChIP-seq: 30 µg of sheared chromatin was used per ChIP reaction. Protein-DNA complexes were incubated with 8 µl antibody against BMAL1 (Abcam, #Ab3350).

Animals and other organisms

Policy information about studies involving animals; ARRIVE guidelines recommended for reporting animal research

Laboratory animals

All mice were group-housed under a 12:12 hr light/dark cycle from birth and fed regular chow (Carfil quality, #10783815) and water ad libitum unless otherwise noted. Mice were maintained at an average temperature of 21 °C and average humidity of 55%.

Wild animals

The study did not involve wild animals.

Field-collected samples

The study did not involve samples collected from the field.

Ethics oversight

All experimental procedures were performed in accordance with the regulatory guidelines of the Ethics Committee for Animal Experimentation from the Medical Sector at UCLouvain (n° LA1230396).

Note that full information on the approval of the study protocol must also be provided in the manuscript.

Human research participants

Policy information about studies involving human research participants

Population characteristics

Obese (class II and III; Body Mass Index BMI >35 kg/m2; 51.8+/- 3.9 yrs) and non-obese patients (BMI <30 kg/m2, with either normal weight or slightly overweight; 50.1+/-3.4 yrs) were age- and sex-matched. Clinical and laboratory characteristics of these patients are summarized in Table 1 (sex ratio is also indicated where appropriate). Patients underwent abdominal surgery after an overnight fast. For non-obese patients, the surgeries included cholecystectomy and treatment of colonic diverticulosis, inguinal hernia or eventration. Obese patients underwent treatments for colonic diverticulosis, eventration or bariatric surgery. Four patients per group were treated for hypertension (two per group with amlodipine, a calcium channel blocker; one per

	group with an angiotensin-converting-enzyme inhibitor; and one per group with bisoprolol, a β1 selective β blocker) and one per group for hypercholesterolemia (statins). Patients with malignancies, receiving hormones (e.g., insulin) or treatments targeting adipose tissue metabolism (e.g., thiazolidinediones) were excluded.<br>Two additional cohorts used for comparison of omental and subcutaneous physiology (Fig.S1) and for in vitro cultures of omental adipocytes (Fig.3H) are described in Table S2.<br>In Cohort 2, paired biopsies of omental and subcutaneous adipose tissue were obtained from 12 obese patients (class III; BMI ≥ 40 kg/m2) undergoing bariatric surgery after an overnight fast. The cohort included 6 men and 6 women. The average age was 36.3± 2.6 years and the average BMI was 45.8 ± 1.6 kg/m2. Two patients were treated for hypertension (amlodipine or angiotensin-converting-enzyme inhibitor) and two for type 2 diabetes (metformin or repaglinide). One patient was treated with a lipase inhibitor.<br>In Cohort 3, omental adipose tissue was obtained from 3 obese patients (BMI ≥ 35 kg/m2) undergoing bariatric surgery after an overnight fast. The cohort included 1 man and 2 women. The average age was 57.7± 2.6 years and the average BMI was 39.5 ± 2.4 kg/m2. All 3 patients had high blood pressure but only two were treated (angiotensin-converting-enzyme inhibitor or angiotensin II receptor antagonist). One patient was treated for type 2 diabetes (metformin) and one for hypothyroidism (L-thyroxine).
Recruitment	Patients who underwent abdominal surgery and who gave informed consent were included at random. There was no selection bias.<br>For non-obese patients, the surgeries included cholecystectomy and treatment of colonic diverticulosis, inguinal hernia or eventration. Obese patients underwent treatments for colonic diverticulosis, eventration or bariatric surgery. Four patients per group were treated for hypertension (two per group with amlodipine, a calcium channel blocker; one per group with an angiotensin-converting-enzyme inhibitor; and one per group with bisoprolol, a β1 selective β blocker) and one per group for hypercholesterolemia (statins). Patients with malignancies, receiving hormones (e.g., insulin) or treatments targeting adipose tissue metabolism (e.g., thiazolidinediones) were excluded.
Ethics oversight	The human study protocol was approved by the local Ethics Committee of Saint-Luc University Hospital (CEHF2017-240)

Note that full information on the approval of the study protocol must also be provided in the manuscript.

ChIP-seq

Data deposition

☒ Confirm that both raw and final processed data have been deposited in a public database such as GEO.

☒ Confirm that you have deposited or provided access to graph files (e.g. BED files) for the called peaks.

Data access links<br>*May remain private before publication.*	GEO repository with the identifier GSE149064.<br>(publicly available, https://www.ncbi.nlm.nih.gov/geo/query/acc.cgi?acc=GSE149064).
Files in database submission	7 samples: GSM4489523 (obeseOAP1), GSM4489524 (obeseOAP2), GSM4489525 (Obese OAP3), GSM4489526 (non-ob OAP1), GSM4489527 (non-ob OAP2), GSM4489528 (non-ob OAP3), GSM4489529 (pooled input DNA)
Genome browser session<br>(e.g. UCSC)	https://genome.ucsc.edu/cgi-bin/hgTracks?hgS_doOtherUser=submit&hgS_otherUserName=eleomaury&hgS_otherUserSessionName=BMAL%20genome%20binding%2Foccupancy%20profiling%20by%20high%20throughput%20sequencing%20in%20human%20mental%20preadipocytes

Methodology

Replicates	OPAs from 3 non-obese and 3 obese (ClassIII) patients who were representative of each group from Table 1
Sequencing depth	Sequencing was generated using 75-bp single-end reads on an Illumina NextSeq 500 instrument to a depth of > 30 million reads. Reads were aligned to the human genome (hg38) using Burrows-Wheeler alignment algorithm with default settings. Only reads that passed Illumina's purity filter, aligned with no more than 2 mismatches, and mapped uniquely to the genome were used in the subsequent analysis. In addition, duplicate reads were removed. For comparative analysis, standard normalization was achieved by down-sampling the usable number of tags (5′-ends of the aligned reads) for each sample in a group (non-obese vs. obese OPAs) to the level of the sample in the group with the fewest usable number of tags. This resulted in an equal number of tags (20,191,577 tags) within each group. The tags were extended in silico using Active Motif software at their 3′- ends to a length of 200 bp (average fragment length in the size selected library bp). To identify the density of fragments (extended tags) along the genome, the genome was divided into 32-nt bins and the number of fragments in each bin was determined. This information was stored in bigWig files for display.
Antibodies	BMAL1 (Abcam, Ab3350, lot Ab GR3285166-5)
Peak calling parameters	Peak calling was performed using the MACS 2.1.0 algorithm with a default cutoff of p value 10-7 for narrow peaks and 10-1 for broad peaks. Peak filtering was performed by removing false ChIP-Seq peaks as defined within the ENCODE blacklist. This process allowed to determine the significant enrichments in the ChIP/IP data file when compared to the Input data file (background).

Data quality	known motifs were identified with the findMotifsGenome program of the HOMER package using default parameters and input sequences comprising +/- 100 bp from the center of the top 1000 peaks. Identification of peaks with statistical enrichment over input across conditions was performed using DESeq2 analysis. The threshold for the number of tags that determines a valid peak was selected for a FDR-adjusted p value of <0.1. This analysis enabled to detect a differential BMAL1 binding genome-wide between non-obese and obese OPAs (Fig.S9).
Software	MACS2.1.0, DESeq2

Flow Cytometry

Plots

Confirm that:

- [x] The axis labels state the marker and fluorochrome used (e.g. CD4-FITC).
- [x] The axis scales are clearly visible. Include numbers along axes only for bottom left plot of group (a 'group' is an analysis of identical markers).
- [x] All plots are contour plots with outliers or pseudocolor plots.
- [x] A numerical value for number of cells or percentage (with statistics) is provided.

Methodology

Sample preparation	Omental adipose tissue was obtained from non-obese and obese patients. Omental adipose tissue was fractionated into adipocytes and stromal-vascular cells by collagenase treatment, as previously described (Maury E, et al., 2007). Omental preadipocytes were obtained from the stromal-vascular fraction after treatment with an erythrocyte lysing buffer, and grown to confluence, as described (Maury E, et al., 2007). After cell dissociation with trypsin followed by trypsin inactivation, omental preadipocytes were washed and resuspended in PBS containing 1% BSA and 1m MEDTA. The cells have been incubated with the antibodies as recommended by the manufacturer's instructions. We used Alexa Fluor® 647 mouse anti-human CD140a BD Biosciences Catalog No: 562798 (clone alphaR1, lot7132680); PE Mouse anti-human CD9 BD Biosciences Catalog No: 555372 (lot 7268691); anti-human CD45-FITC Immunotools Catalog No: 21810453X2 (lot Sp034); anti-human CD31-FITC Miltenyi Biotec Catalog No: 130-117-539.<br>(For note, the axis labels state the marker but the fluorochrome used is reported in the legend).<br>Initial experiments aimed at optimizing the dissociation for flow cytometry analysis and at testing the antibodies were performed using a FACSCalibur flow cytometer and CellQuest software v5.
Instrument	FACSCanto II BD Biosciences
Software	FlowJo v10 BD Biosciences ; FACS DIVA v8
Cell population abundance	CD140a (PDGFRa)/ CD9 positive cells isolated from omental adipose tissue were analyzed (double staining). For each patient, unstained cells have been used as negative controls for each marker. Approximately 97.5-98% of these cells were PDGFRa positive with no significant difference between non-obese and obese patients, while the percentage of CD9 positive cells represented 75% to 96% of these cells. In a separate experiment, these cells have been stained with either anti-human CD45-FITC or anti-human CD31-FITC, and unstained cells have been used as negative controls for each staining. These two markers (CD45 and CD31) were not detected in the omental adipose precursors. APC/Cyanine7 mouse anti-human CD34 BioLegend Catalog No: 343613 was detected in PGDF positive cells.
Gating strategy	FSC vs. SSC to identify the cells (as described in Brusa and Balligand, 2019). CD140/ CD9/CD34 dot plots were used to identify the populations of interest. Please see Fig S8.

- [x] Tick this box to confirm that a figure exemplifying the gating strategy is provided in the Supplementary Information.
